# Prognostic impact of clinicopathologic parameters in stage II/III breast cancer treated with neoadjuvant docetaxel and doxorubicin chemotherapy: paradoxical features of the triple negative breast cancer

**DOI:** 10.1186/1471-2407-7-203

**Published:** 2007-11-01

**Authors:** Bhumsuk Keam, Seock-Ah Im, Hee-Jun Kim, Do-Youn Oh, Jee Hyun Kim, Se-Hoon Lee, Eui Kyu Chie, Wonshik Han, Dong-Wan Kim, Woo Kyung Moon, Tae-You Kim, In Ae Park, Dong-Young Noh, Dae Seog Heo, Sung Whan Ha, Yung-Jue Bang

**Affiliations:** 1 Department of Internal Medicine, Seoul National University College of Medicine, Seoul, Korea; 2 Department of Radiation Oncology, Seoul National University College of Medicine, Seoul, Korea; 3 Department of Surgery, Seoul National University College of Medicine, Seoul, Korea; 4 Department of Radiology, Seoul National University College of Medicine, Seoul, Korea; 5 Department of Pathology, Seoul National University College of Medicine, Seoul, Korea; 6 Cancer Research Institute, Seoul National University College of Medicine, Seoul, Korea

## Abstract

**Background:**

Prognostic factors in locally advanced breast cancer treated with neoadjuvant chemotherapy differ from those of early breast cancer. The purpose of this study was to identify the clinical significance of potential predictive and prognostic factors in breast cancer patients treated by neoadjuvant chemotherapy.

**Methods:**

A total of 145 stage II and III breast cancer patients received neoadjuvant docetaxel/doxorubicin chemotherapy were enrolled in this study. We examined the clinical and biological factors (ER, PR, p53, c-erbB2, bcl-2, and Ki-67) by immunohistochemistry. We analyzed clinical outcome and their correlation with clinicopathologic parameters.

**Results:**

Among the clinicopathologic parameters investigated, none of the marker was correlated with response rate (RR) except triple negative phenotype. Patients with triple negative phenotype showed higher RR (83.0% in triple negative *vs*. 62.2% in non-triple negative, *p *= 0.012) and pathologic complete RR (17.0% in triple negative *vs*. 3.1% in non-triple negative, *p *= 0.005). However, relapse free survival (RFS) and overall survival (OS) were significantly shorter in triple negative breast cancer patients (*p *< 0.001, *p *= 0.021, respectively). Low histologic grade, positive hormone receptors, positive bcl-2 and low level of Ki-67 were associated with prolonged RFS. In addition, positive ER and positive bcl-2 were associated with prolonged OS. In our homogeneous patient population, initial clinical stage reflects RFS and OS more precisely than pathologic stage. In multivariate analysis, initial clinical stage was the only significant independent prognostic factor to impact on OS (hazard ratio 3.597, *p *= 0.044).

**Conclusion:**

Several molecular markers provided useful predictive and prognostic information in stage II and III breast cancer patients treated with neoadjuvant docetaxel/doxorubicin chemotherapy. Triple negative phenotype was associated with shorter survival, even though it was associated with a higher response rate to neoadjuvant chemotherapy.

## Background

Neoadjuvant chemotherapy has become a standard therapy for patients with locally advanced breast cancer [1,2]. Major roles of neoadjuvant chemotherapy are 1) conversion of inoperable or inflammatory breast cancer to operable status 2) increasing the rate of breast conserving surgery, and 3) individual *in vivo *chemosensitivity test of the tumor [2-4]. However, a potential disadvantage of neoadjuvant chemotherapy is the loss of prognostic value provided by tumor size and nodal status at surgery and before adjuvant chemotherapy [3,4].

A number of studies have investigated prognostic factors in the neoadjuvant setting. At present, pathologic complete response (pCR) is a useful independent prognostic factor and the patients who achieved pCR showed better survival compared with those with residual tumor [5-8]. However a small percentage of patients achieved pCR, and a significant portion of patients with pCR had recurrent disease [9]. Molecular markers such as estrogen receptor (ER), progesterone receptor (PR), p53, Ki-67 and c-erbB2 considered predictive or prognostic factors in neoadjuvant setting [7,10-14]. However, these markers are often contradictory and not conclusive because of heterogeneous patient populations, small sample sizes, and different chemotherapeutic regimens. Due to alterations in molecular mechanism during neoadjuvant chemotherapy, and also uncertainty regarding the prognostic value of clinicopatholgic parameters, physicians felt difficulties to accurately define risk profiles and identify optimal post operation treatment including chemotherapy and radiation therapy.

We ourselves have conducted neoadjuvant docetaxel/doxorubicin combination chemotherapy in stage II and III breast cancer patients. The purpose of this study was to identify the clinical significance of potential predictive and prognostic factors in the neoadjuvant setting.

## Methods

### Patients and treatment

From March 2002 to March 2006, patients were enrolled in this study. Eligibility criteria included: 1) pathologically confirmed breast cancer by core needle biopsy, 2) clinical stage II or III, 3) objective measurable lesion, 4) ECOG performance 0–2, 5) previously untreated, 6) adequate bone marrow, hepatic, cardiac, and renal functions. Initial evaluation included clinical examination, mammography, breast ultrasonography, computed tomography of chest, bone scan, and breast magnetic resonance imaging (MRI). Initial tumor size was measured by MRI. Initial nodal staging was evaluated by physical examination and by computed tomography. After three cycles of neoadjuvant chemotherapy, the patients were re-evaluated for response.

The chemotherapeutic regimen comprised docetaxel (75 mg/m^2 ^or 60 mg/m^2^) and doxorubicin (60 mg/m^2 ^or 50 mg/m^2^) by intravenous infusion every three weeks for three cycles, with granulocyte colony stimulating factor as primary prophylaxis. After completion of neoadjuvant treatment, the patients underwent primary surgery and received three more cycles of docetaxel and doxorubicin as adjuvant chemotherapy, followed by radiation or hormonal therapy if indicated [15]. If the patients had been found to have progressive disease, they underwent primary surgery and received adjuvant chemotherapy using different regimens. This regimen was known to be effective and well tolerated as neoadjuvant chemotherapy for stage II or III breast cancer [16].

Radiologic response was evaluated using breast MRI for primary breast cancer measurement and chest CT for lymph node measurement by RECIST criteria [17] as follows; complete response was defined as the complete disappearance of all assessable lesions; partial response as a >30% reduction in the sum of the longest diameters of all measurable lesions; stable disease as a <30% reduction or a <20% increase in the sum of the longest diameters of all measurable lesions; and progressive disease was defined as >20% increase in the area(s) of original measurable lesion or the appearance of a new lesion.

We examined the conventional clinicopathologic factors including the six different biological factors (ER, PR, p53, c-erbB2, bcl-2, and Ki-67) by immunohistochemistry and evaluated their association with clinical outcomes. The study protocol was reviewed and approved by the institutional review board at the Seoul National University Hospital. Recommendations of the Declaration of Helsinki for biomedical research involving human subjects were also followed.

### Pathologic Examination and Immunohistochemistry

The pretreatment formalin-fixed, paraffin-embedded tissue blocks were used for immunohistochemistry. The pathological tumor stage assessed according to the criteria established by the 6th edition of AJCC cancer staging manual [18], the grade of the tumor according to the Scarff-Bloom-Richardson classification modified by Elston and Ellis [19]. The pathologic complete response (pCR) was defined as complete disappearance of invasive carcinoma in both breast and axillary lymph nodes after three cycles of chemotherapy. Residual ductal carcinoma *in-situ *was included in the pCR category.

ER, PR, c-erbB2, p53, bcl-2, and Ki-67 expressions were evaluated by the avidin-biotin complex immunohistochemical technique [20]. Tissue sections (4-μm thickness) from paraffin-embedded tissue blocks were cut, deparaffinized in xylene, rehydrated with graded ethanol, and immersed in Tris-buffered saline. After an antigen-retrieval process, primary antibodies were used as previously described [21]. The companies that supplied the primary antibodies and the dilution factors used were; ER (Dako Corporation, Carpinteria, CA, USA; 1:50), PR (Dako Corporation; 1:50), c-erbB2 (Novocastra Laboratories Ltd., New Castle-Upon-Tyne, U.K.; 1:200), p53 (Dako Corporation; 1:1200), bcl-2 (Dako Corporation; 1:50), and Ki-67 (Dako Corporation; 1:800). All primary antibodies were mouse monoclonal antibodies. Biotinylated anti-mouse antibody was used as secondary antibody and streptavidin horseradish peroxidase (Zymed laboratories, San Francisco, CA, USA) methods were used.

A cut-off value of 10% or more positively stained nuclei in ten high-power fields was used to define ER and PR positivity. C-erbB2 scores of 0, 1 and 2 were considered negative, and a score of 3 was considered positive [22]. In the current study, we did not have FISH information available on the majority of c-erbB2 positive patients. Ki-67 with ≤ 5% and p53 with <25% were considered as low expression. Triple negative subtype was defined as ER negative, PR negative, and c-erbB2 negative, regardless of the expression of EGFR or basal cytokeratins.

### Statistical analysis

The significance of the difference in the response rate among different groups was calculated using the Chi-squared test and Fisher's exact test, where appropriate. Multivariate analyses were carried out using the Cox proportional hazard regression models. Relapse free survival (RFS) was determined as the interval between the initiation of neoadjuvant chemotherapy and the date when disease relapse or progression was first documented or the date of death from any cause. Overall survival (OS) was measured from the date of neoadjuvant chemotherapy initiation to the date of death. Survival comparisons between different groups were made using the log-rank tests. All values were two sided and statistical significance was defined as *p *< 0.05. SPSS version 12.0 software (SPSS, Inc., Chicago, IL, USA) was used for all statistical analyses.

## Results

### Patient Characteristics and efficacy

A total of 145 patients with a median age of 45 (range 25–69) were evaluated in this study. The clinical characteristics of the patients are summarized in Table 1. Most of the patients (84.1%) were clinical stage III at the time of initial diagnosis and eighteen patients (12.4%) had inflammatory breast cancers. The median tumor size was 5 cm which is relatively large for Asian woman who have small breast. The breast conserving surgery rate was 35.9%. The overall radiologic response rate (RR) was 68.9% including 7 complete response (4.8%) and 93 partial response (64.1%) (Table 2). All 7 radiologic complete responder showed pCR and four patients who showed radiologic residual lesion were turned out to pCR. Consequently, eleven patients (7.6%) achieved a pCR (Table 2).

**Table 1:** Clinical characteristics of 145 patients

Characteristics	No. of Pt (%)
Median age (range)	45 (range 25–69)
Age < 50	102 (70.3)
Age ≥ 50	43 (29.7)
Performance status	
ECOG 0–1	139 (95.9)
ECOG 2	6 (4.1)
Pathologic characteristics	
Invasive ductal carcinoma	137 (94.5)
Others	8 (5.5)
Initial clinical stage	
IIA	2 (1.4)
IIB	21 (14.5)
IIIA	70 (48.3)
IIIB	34 (23.4)
IIIC	18 (12.4)
Median tumor size	5.0 cm (range 1.2–12.0 cm)
Inflammatory breast cancer	
Yes	18 (12.4)
No	127 (87.6)
Type of surgery	
Breast conserving	52 (35.9)
Mastectomy	93 (64.1)
Adjuvant hormonal therapy	
Yes	63 (43.4)
No	82 (56.6)
Radiation therapy	
Yes	128 (88.3)
No	17 (11.7)

ECOG, Eastern Cooperative Oncology Group.

**Table 2:** Radiologic and pathologic response after docetaxel plus doxorubicin neoadjuvant chemotherapy

Response	No. of Pts (%)
Radiologic response	
Complete response	7 (4.8)
Partial response	93 (64.1)
Stable disease	42 (29.0)
Progressive disease	3 (2.1)
Pathologic complete response	
Yes	11 (7.6)
No	134 (92.4)

Of 145 patients, 138 patients including patients with pCR received three more cycles of docetaxel and doxorubicin as planned adjuvant chemotherapy. Three patients who showed progressive disease and 4 patients who were unacceptable to docetaxel received different adjuvant chemotherapy using FAC (5-fluorouracil, doxorubicin, cyclophosphamide), AC (doxorubicin, cyclophosphamide) or CMF (cyclophosphamide, methotrexate, 5-fluorouracil) after curative surgery.

Median follow-up duration was 18.6 months. Estimated one and three year relapse free survival rates were 88.7% and 56.5%, respectively. Estimated one and three year overall survival rates were 97.5% and 71.6% respectively.

### Correlation between clinicopathological variables and response rate

Potential traditional predictive factors (age, performance, stage, nuclear grade, histologic grade, ER, PR, p53, c-erbB2, bcl-2 and Ki-67) were analyzed. Table 3 compares radiologic RR and predictive factors. pCR was correlated with radiologic RR (*p *= 0.018). pCR and radiologic RR according to ER/PR/c-erbB2 are summarized in Table 4. Patients with triple negative breast cancer showed higher RR (83.0% in triple negative *vs*. 62.2% in non-triple negative, *p *= 0.012).

**Table 3:** Correlation between clinicopathological variables and radiologic response rate

Variables		No. of Pts	Responders (RR %)	p-value*
Age	< 50 ≥ 50	102 43	69 (67.6) 31 (72.1)	0.597
Performance	ECOG 0–1 ECOG 2	139 6	95 (68.3) 5 (83.3)	0.666
Initial clinical stage	IIA, IIB, IIIA IIIB, IIIC	93 52	64 (68.8) 36 (69.2)	0.959
pCR	No	134	89 (66.4)	0.018
	Yes	11	11 (100.0)	
Nuclear grade	I, II	41	22 (53.7)	0.069
	III	87	61 (70.1)	
	Unknown	17	-	
Histologic grade	I, II	39	24 (61.5)	0.741
	III	82	53 (64.6)	
	Unknown	24	-	
ER	Positive	64	41 (64.1)	0.257
	Negative	81	59 (72.8)	
PR	Positive	44	28 (63.6)	0.360
	Negative	101	72 (71.3)	
bcl-2	Positive	63	45 (71.4)	0.749
	Negative	74	51 (68.9)	
	Unknown	8	-	
Ki-67	Low expression^#^	56	34 (60.7)	0.066
	High expression	85	64 (75.3)	
	Unknown	4	-	
p53	Low expression^#^	67	43 (64.2)	0.219
	High expression	76	56 (73.7)	
	Unknown	2	-	
c-erbB2	0/+/++	107	76 (71.0)	0.368
	+++	38	24 (63.2)	
Triple negative	No	98	61 (62.2)	0.012
	Yes	47	39 (83.0)	

RR, response rate; pCR, pathologic complete response; ER, estrogen receptor; PR, progesterone receptor.

*based on Pearson's χ^2 ^test (using Fisher's exact test if N ≤ 5).

^#^Ki-67 with ≤ 5% and p53 with <25% were considered as low expression.

**Table 4:** Pathologic complete response and radiologic response rate according to ER/PR/c-erbB2

		No. of Pts	pCR (N = 11)	*p-value*	Radiologic Response (RR %)	*p-value*
			
Variables			No. of Pts (%)		No. of Pts (%)	
ER	Positive	64	1 (1.6)	0.023	41 (64.1)	0.257
	Negative	81	10 (12.3)		59 (72.8)	
PR	Positive	44	1 (2.3)	0.173	28 (63.6)	0.360
	Negative	101	10 (9.9)		72 (71.3)	
c-erbB2	0/+/++	107	9 (8.4)	0.728	76 (71.0)	0.368
	+++	38	2 (5.3)		24 (63.2)	

### Correlation between clinicopathological variables and survival

**Figure 1:**
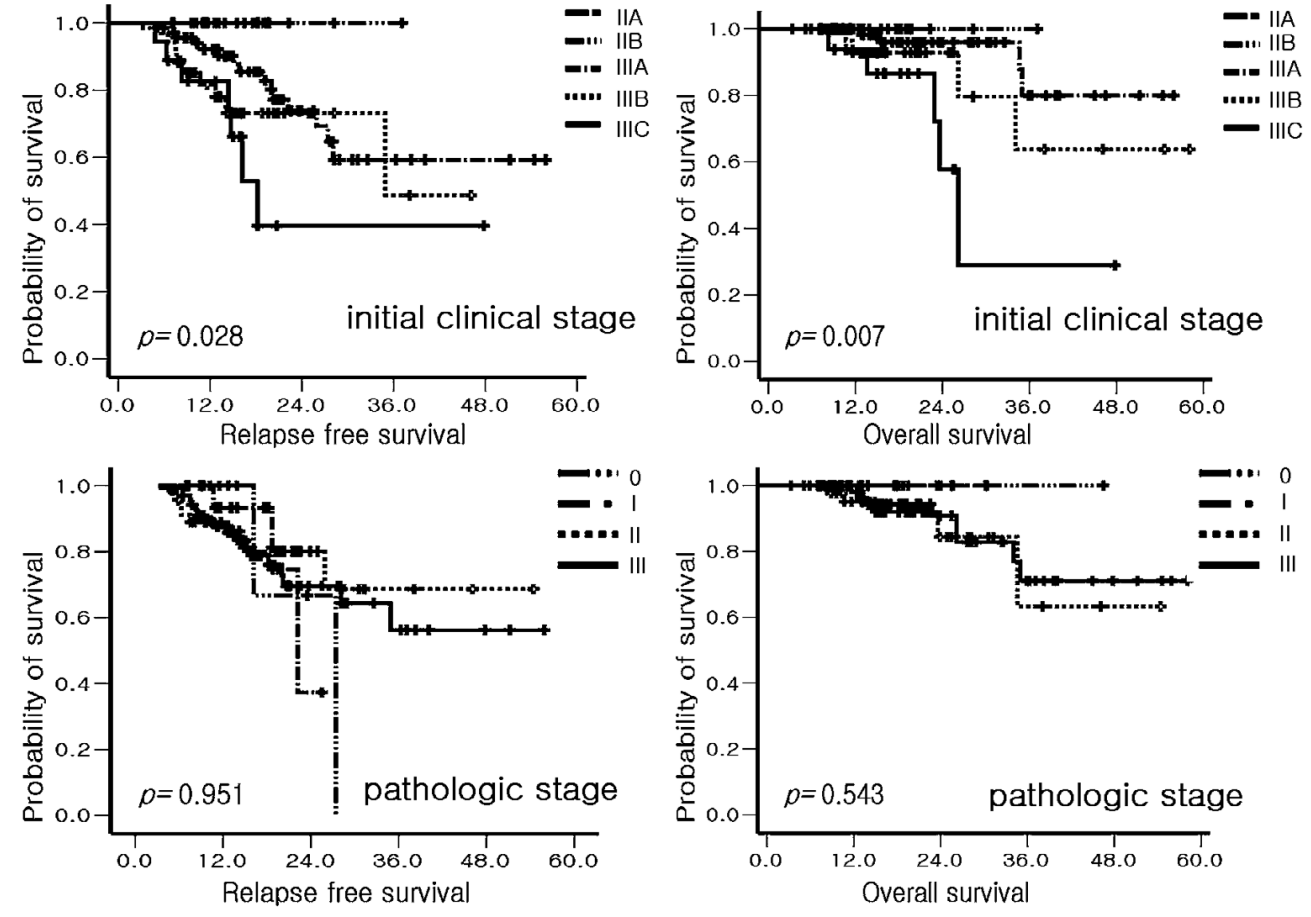
Kaplan-Meier analyses of survival according to clinical and pathologic stages.

The results of univariate analyses for RFS and OS were shown in Table 5. Responding to neoadjuvant chemotherapy did not affect RFS or OS. Among the parameters investigated, low histologic grade, positive ER, positive PR, positive bcl-2 and low level of Ki-67 were associated with prolonged RFS in univariate analysis. In addition, positive ER and positive bcl-2 were associated with prolonged overall survival (OS) in univariate analysis. In terms of stage, initial clinical stage reflects RFS and OS more precisely than pathologic stage. Kaplan-Meier plots (Figure 1) show the survival curve according to clinical and pathologic stage.

**Table 5:** Correlation between clinicopathological variables and survival-univariate analysis

			RFS		OS	
			
Variables		No. of Pt	HR* (95% CI)	*p-value*	HR* (95% CI)	*p-value*
Age	<50	102	1	0.317	1	0.283
	≥50	43	1.476 (0.688–3.166)		1.831 (0.606–5.533)	
Performance	ECOG 0–1	139	1	0.686	1	0.888
	ECOG 2	6	1.349 (0.316–5.756)		1.160 (0.148–9.073)	
Initial clinical stage	IIA, IIB, IIIA	93	1	0.017	1	0.010
	IIIB, IIIC	52	2.370 (1.116–4.815)		4.764 (1.462–15.525)	
pCR	No	134	1	0.817	NA^#^	NA^#^
	Yes	11	1.186 (0.281–5.005)			
Pathologic stage	pCR~IIIA	113	1	0.288	1	0.086
	IIIB, IIIC	32	1.525 (0.700–3.319)		2.608 (0.874–7.786)	
Pathologic N stage	N0	42	1	0.636	1	0.566
	N1–3	103	0.828 (0.378–1.812)		1.558 (0.342–7.099)	
Radiologic response	Responder	100	1	0.515	1	0.683
	Non-responder	45	0.776 (0.361–1.665)		1.258 (0.419–3.775)	
Nuclear grade	I, II	41	1	0.151	1	0.141
	III	87	1.894 (0.792–4.532)		2.689 (0.722–10.023)	
Histologic grade	I, II	39	1	0.020	1	0.132
	III	82	4.159 (1.248–13.865)		4.820 (0.621–32.387)	
ER	Positive	64	1	0.001	1	0.028
	Negative	81	5.410 (2.073–14.119)		9.921 (1.289–76.349)	
PR	Positive	44	1	0.005	1	0.166
	Negative	101	7.778 (1.851–32.673)		4.278 (0.547–33.476)	
bcl-2	Positive	63	1	0.034	1	0.046
	Negative	74	2.351 (1.068–5.175)		4.705 (1.030–21.490)	
Ki-67	Low expression	56	1	0.038	1	0.082
	High expression	85	2.357 (1.050–5.287)		3.263 (0.861–12.363)	
p53	Low expression	67	1	0.869	1	0.670
	High expression	76	1.063 (0.515–2.193)		1.281 (0.410–3.998)	
c-erbB2	0/+/++	107	1	0.242	1	0.678
	+++	38	1.555 (0.742–3.255)		1.273 (0.408–3.973)	
Triple negative	No	98	1	0.002	1	0.029
	Yes	47	3.148 (1.539–6.441)		3.430 (1.133–10.378)	

RFS, relapse free survival; OS, overall survival; HR, hazard ratio; CI, confidence interval.

*Hazard ratio was calculated by Cox's proportional hazard model. If the hazard ratio is greater than 1, the hazard ratio can be thought of as the average increased risk of relapse or dying at any point in time compared with the reference group (described upper line).

^#^NA: Not available due to all censored in pCR.

We also performed multivariate analysis (Table 6). Cox proportional hazard regression analysis for OS included statistically significant variables (initial clinical stage, ER, bcl-2, and triple negative). In multivariate analysis, initial clinical stage was the only significant independent prognostic factor to impact on OS (hazard ratio 3.597, *p *= 0.044).

**Table 6:** Multivariate Cox regression analyses for the factors associated with overall survival

	OS		
	
Variables	HR	95% CI	*p*-value
Initial clinical stage	3.597	1.037–12.480	0.044
ER	3.329	0.296–37.454	0.330
bcl-2	3.027	0.557–16.437	0.200
Triple negative	1.847	0.492–6.935	0.364

### Clinical significance of triple negative breast cancer

**Figure 2:**
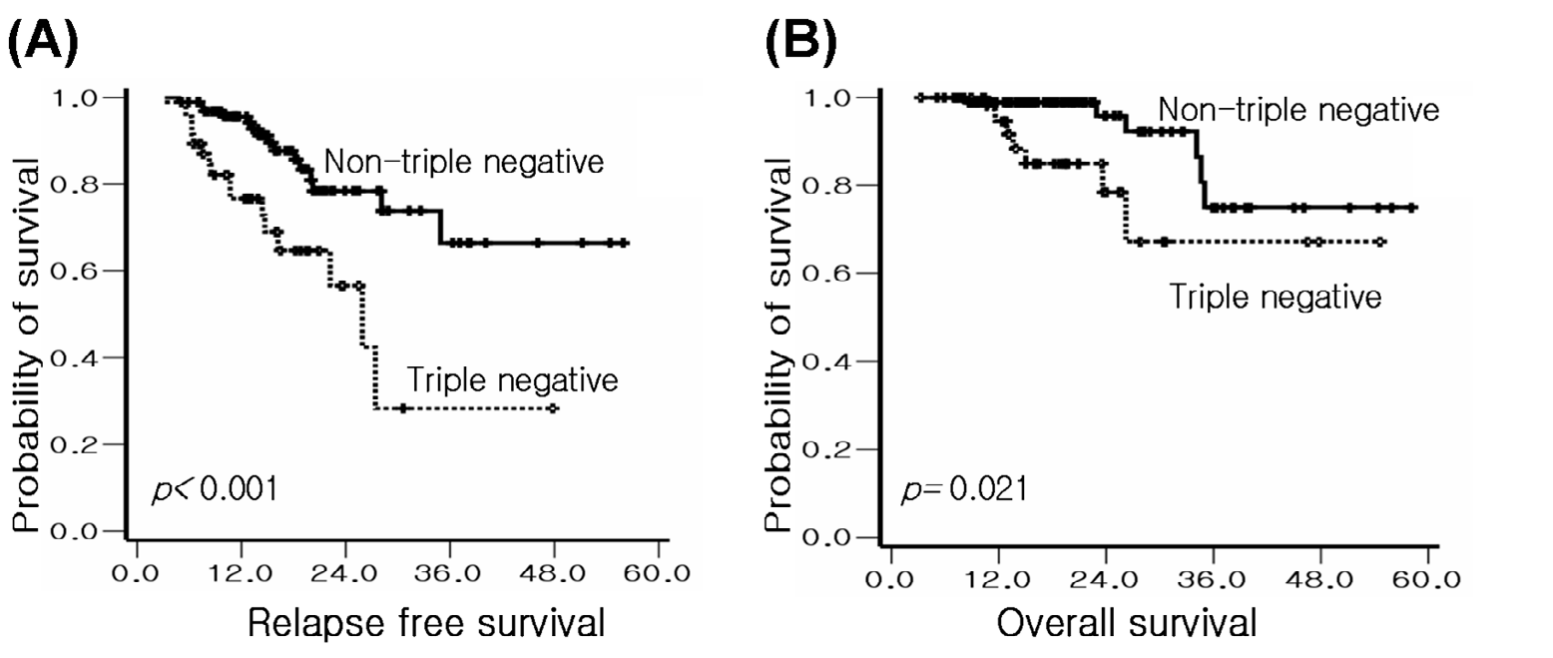
Kaplan-Meier analyses of (A) relapse free and (B) overall survival according to triple negative and non-triple negative breast cancer.

Forty seven patients (32.4%) of the 145 were triple negative breast cancer. Clinicopathologic variables according to triple negative are summarized in Table 7. Triple negative breast cancer patients showed statistically higher nuclear grade, and lower bcl-2 positive rate than non-triple negative breast cancer patients. A trend for high levels of Ki-67 was also observed in triple negative, although it did not reach statistical significance (*p *= 0.053). The pCR rate and clinical RR in triple negative were significantly higher (*p *= 0.005, *p *= 0.012, respectively). However, RFS and OS were significantly short in triple negative breast cancer patients (*p *< 0.001, *p *= 0.021, respectively). RFS and OS survival curves for triple negative and non-triple negative are shown in Figure 2. Because c-erbB2 positivity by immunohistochemistry was unclear, we conducted a second analysis considering 2+ as c-erbB2 positive. Using this definition of triple negative, the results were similar.

**Table 7:** Clinicopathologic characteristics according to triple negative and non-triple negative breast cancer

		No. of Pts	Triple negative (N = 47)	Non-triple negative (N = 98)	p-value*
			
Variables			No. of Pts (%)	No. of Pts (%)	
Age	< 50	102	33 (70.2)	69 (70.4)	0.981
	≥ 50	43	14 (29.8)	29 (29.6)	
Performance	ECOG 0–1	139	45 (95.7)	94 (95.9)	0.961
	ECOG 2	6	2 (4.3)	4 (4.1)	
Initial clinical stage	IIA, IIB, IIIA	93	27 (57.4)	66 (67.3)	0.245
	IIIB, IIIC	52	20 (42.6)	32 (32.7)	
Inflammatory breast cancer	Yes	18	4 (8.5)	14 (14.3)	0.425
	No	127	43 (91.5)	84 (85.7)	
Radiologic response	Responder	100	39 (83.0)	61 (62.2)	0.012
	Non-responder	45	8 (17.0)	37 (37.8)	
pCR	No	134	39 (83.0)	95 (96.9)	0.005
	Yes	11	8 (17.0)	3 (3.1)	
Adjuvant hormonal therapy	Yes	63	0 (0.0)	63 (64.3)	<0.001
	No	82	47(100.0)	35 (35.7)	
Radiation therapy	Yes	128	41 (87.2)	87 (88.8)	0.787
	No	17	6 (12.8)	11 (11.2)	
Nuclear grade	I, II	41	3 (8.1)	38 (41.8)	<0.001
	III	87	34 (91.9)	53 (58.2)	
Histologic grade	I, II	39	7 (20.0)	32 (37.2)	0.066
	III	82	28 (80.0)	54 (62.8)	
bcl-2	Positive	63	14 (31.8)	49 (52.7)	0.022
	Negative	74	30 (68.2)	44 (47.3)	
Ki-67	Low expression	56	13 (28.3)	43 (45.3)	0.053
	High expression	85	33 (71.7)	52 (54.7)	
p53	Low expression	67	22 (46.8)	45 (46.9)	0.994
	High expression	76	25 (53.2)	51 (53.1)	

*based on Pearson's χ^2 ^test (using Fisher's exact test if N ≤ 5)

## Discussion

The clinical course of breast cancer patients treated with neoadjuvant chemotherapy remains difficult to predict, because histologically homogeneous breast cancers vary in response to therapy and have divergent outcomes [23]. As a result, many researchers have tried to identify prognostic factors in order to give optimal individualized therapy in locally advanced breast cancer, as well as in early breast cancer. Currently, pCR is the most powerful prognostic factor for prolonged survival in neoadjuvant chemotherapy [3,5,6,24]. However, a significant proportion of patients with pCR have recurrent diseases [9]. Moreover, the prognostic factors for patients receiving neoadjuvant chemotherapy differ from those for patients who receive adjuvant or palliative chemotherapy, because pathologic parameters including tumor size and nodal status are changed by neoadjuvant chemotherapy [3]. Hence we tried to determine the additional predictive and prognostic markers for early relapse other than pCR in neoadjuvant setting.

In the present study, we found that a triple negative phenotype was a predictive marker for response in neoadjuvant docetaxel and doxorubicin chemotherapy. In addition, initial clinical stage, hormone receptor, histologic grade, bcl-2 and Ki-67 were all associated with RFS. In other published studies using non-anthracycline based chemoendocrine agents [11], it was reported that positive ER, absence of c-erbB2 and decrease in Ki-67 were associated with a good clinical response. Overexpression of p53 was also reported to be associated with a lower response rate to anthracycline based neoadjuvant chemotherapy [13,14,25] and to be an independent factor for poor survival [14,25]. In our results, overexpression of p53 failed to show clinical significance in neoadjuvant setting. However, p53 mutation which was associated with response to neoadjuvant chemotherapy [13] was not in agreement with p53 overexpression measured by quantitative immunohistochemistry. Additional mutational study of p53 is needed to clarify correlation between p53 and clinical outcomes. The predictive or prognostic value of bcl-2, apoptosis regulatory protein, remains controversial in neoadjuvant setting. In one study, higher bcl-2 expression was predictive for pCR [26], while other studies did not find any correlation between bcl-2 expression and clinical response [25,27]. Traditional prognostic makers such as nodal stage [28] and c-erbB2 [10,11] showed no prognostic value in our result. Relatively short follow up period of 18.6 months might partially explain this. As yet, these biologic markers are inconclusive, owing to heterogeneous chemotherapeutic regimens and the small sample size of extant studies. More studies should be carried out, to identify more precisely the prognostic markers in the neoadjuvant setting.

In our results, pCR which is considered to be the most powerful prognostic factor did not show significant prognostic value. Possible explanations for the weakened prognostic power of pCR are the relatively lower rate of pCR (7.6%), the short course of neoadjuvant chemotherapy, and the short duration of follow up (18.6 months). We conducted only three cycles of neoadjuvant chemotherapy, while other neoadjuvant regimens have been based on four to six cycles, and have shown higher pCR rates (8–26%) than our own study [24,29,30].

Optimal treatment after neoadjuvant chemotherapy remains still uncertain [31]. Unlike early breast cancer, it is not yet clear whether adjuvant therapy should be conducted according to initial clinical stage or post operative pathologic stage. In our homogeneous patient population, initial clinical stage was an independent prognostic factor for survival, while pathologic stage failed to reflect ultimate survival. This result was obtained by using accurate staging work up modalities, including breast MRI and chest computed tomography. In contrast, Carey et al [32] analyzed 135 patients with median follow up of 5 years and reported that pathologic stage after neoadjuvant chemotherapy was useful for predicting survival. Chollet et al [33] also reported prognostic value of residual tumor size and nodal status after neoadjuvant chemotherapy with median follow up of 9.3 year. However, despite short duration of follow up, our results showed statistical superiority of initial clinical stage in predicting survival. This result might give us useful information when determining post operative adjuvant therapy.

Triple negative breast cancer has been reported as being associated with a poor clinical outcome in early breast cancer [34,35]. In locally advanced breast cancer, there are limited data about response to chemotherapy and survival. In the present study, we found that triple negative breast cancer responded to neoadjuvant chemotherapy initially but then relapsed rapidly. Generally, tumor responsiveness to chemotherapy is believed to be associated with a longer survival. However, in triple negative phenotype, tumor responsiveness did not affect prolonged survival. In contrast, non-triple negative breast cancer did not show a marked response but progressed rather slowly. This paradoxical feature is consistent with other studies conducted in basal-like breast cancer, which was identified using gene expression profiling [36,37]. In the present study, we did not conduct gene expression profiling and hierachial cluster analysis. However, it is known that 80–90% of triple negative breast cancers by immunohistochemistry are basal-like subtypes by gene expression profiling [35] and have a similar clinical behavior, in addition [38,39]. It is notable that we were able to obtain useful predictive and prognostic information by simple immunohistochemistry without high cost.

It is not yet certain whether the poor prognosis of triple negative breast cancer is due to its aggressive feature or because of lack of targeted therapy, including adjuvant hormonal therapy and c-erbB2 targeted agents. We hypothesized that triple negative breast cancer itself seems to reflect more aggressive tumor biology and growth rate potential with high expression of Ki-67. Our data suggest that patients with triple negative breast cancer should be candidates for clinical trials to determine additional agents including antiangiogenic agents.

## Conclusion

Several molecular markers play a role as predictive and prognostic factors in stage II and III breast cancer patients receiving neoadjuvant chemotherapy. We also confirmed the usefulness of initial clinical stage, as a predictor of survival. We found that triple negative phenotype was associated with shorter survival, even though it was associated with a higher response rate to neoadjuvant chemotherapy. These results might assist in identifying and understanding the importance of clinically useful markers in the neoadjuvant setting, and help to optimize treatments.

## Competing interests

The author(s) declare that they have no competing interests.

## Authors' contributions

BK collected the data, performed the statistical analysis and drafted the manuscript. SAI designed the concept of this study, performed the statistical analysis with interpretation and approved the final manuscript. HJK collected the data. DYO, JHK, SHL, DWK, TYK, DSH and YJB performed the chemotherapy for patients and revised the manuscript. EKC and SWH performed radiation therapy for patients and participated in treatment coordination. WH and DYN performed operation and treatment coordination. WKM reviewed the breast images and measured the tumor size. IAP carried out the immunoassays and pathologic examinations. All authors read and approved the final manuscript.

## Pre-publication history

The pre-publication history for this paper can be accessed here:


